# Cytotoxic effect of different treatment parameters in pressurized intraperitoneal aerosol chemotherapy (PIPAC) on the in vitro proliferation of human colonic cancer cells

**DOI:** 10.1186/s12957-017-1109-4

**Published:** 2017-02-10

**Authors:** Veria Khosrawipour, David Diaz-Carballo, Acikelli Ali-Haydar, Tanja Khosrawipour, Thomas Albert Falkenstein, Dan Wu, Jürgen Zieren, Urs Giger-Pabst

**Affiliations:** 1 Department of General Surgery and Therapy Center for Peritonealcarcinomatosis, St. Mary’s Hospital Herne, Ruhr University of Bochum, Hölkeskampring 40, 44625 Herne, Germany; 2 Basic Research Laboratory Department of Surgery, St. Mary’s Hospital Herne, Ruhr University of Bochum, Herne, Germany; 3 Department of Hematology and Medical Oncology, St. Mary’s Hospital Herne, Ruhr University Bochum, Herne, Germany

**Keywords:** In vitro model, Human colonic cancer cells, PIPAC, Cytotoxicity, Pressure, Oxaliplatin dose escalation

## Abstract

**Background:**

Pressurized intraperitoneal aerosol chemotherapy (PIPAC) has been recently reported as a new approach for intraperitoneal chemotherapy (IPC). By means of a patented micropump, the liquid chemotherapy is delivered into the peritoneal cavity as an aerosol which is supposed to achieve “gas-like” distribution. However, recent data report that the fraction of the submicron aerosol (gas-like) is less than 3 vol% of the total amount of aerosolized chemotherapy. Until today, possible modifications of treatment parameters during PIPAC with the aim of improving therapeutic outcomes have not been studied yet.

This study aims to establish an in vitro PIPAC model to explore the cytotoxic effect of the submicron aerosol fraction and to investigate the impact of different application parameters on the cytotoxic effect of PIPAC on human colonic cancer cells.

**Methods:**

An in vitro model using HCT8 colon adenocarcinoma wild-type cells (HCT8^WT^) and multi-chemotherapy refractory subline (HCT8^RT^) was established. Different experimental parameters such as pressure, drug dosage, time exposure, and system temperature were monitored in order to search for the conditions with a higher impact on cell toxicity. Cell proliferation was determined by means of colorimetric MTT assay 48 h following PIPAC exposures.

**Results:**

Standard operational parameters applied for PIPAC therapy depicted a cytotoxic effect of the submicron aerosol fraction generated by the PIPAC micropump. We also observed that increasing pressure significantly enhanced tumor cell toxicity in both wild-type and chemotherapy-resistant cells. A maximum of cytotoxicity was observed at 15 mmHg. Pressure >15 mmHg did not show additional cytotoxic effect on cells. Increased oxaliplatin dosage resulted in progressively higher cell toxicity as expected. However, in resistant cells, a significant effect was only found at higher drug concentrations. Neither an extension of exposure time nor an increase in temperature of the aerosolized chemotherapy solution added an improvement in cytotoxicity.

**Conclusions:**

In this in vitro PIPAC model, the gas-like PIPAC aerosol fraction showed a cytotoxic effect which was enhanced by higher intra-abdominal pressure with a maximum at 15 mmHg. Similar findings were observed for drug dose escalation. A phase I dose escalation study is currently performed at our institution. However, increasing the intra-abdominal pressure might be a first and simple way to enhance the cytotoxic effect of PIPAC therapy which needs further clinical investigations.

**Electronic supplementary material:**

The online version of this article (doi:10.1186/s12957-017-1109-4) contains supplementary material, which is available to authorized users.

## Background

The survival benefit of intraperitoneal chemotherapy (IPC) was first demonstrated more than 20 years ago and has since then been confirmed in multiple additional trials. However, inadequate drug delivery to solid tumors is a major reason for treatment failure in systemic and IPC strategies [1, 2]. One of the assumed reasons is an increased intratumoral pressure hampering the convective influx of anticancer drugs into the tumoral tissue as opposed to normal tissue [3].

Pressurized intraperitoneal aerosol chemotherapy (PIPAC) has been recently reported as a new approach for IPC which could overcome the outlined limitations of IPC with liquid solutions. Using a micropump, the drug containing solution is delivered into the abdominal cavity in the shape of micro droplets within the 12 mmHg capnoperitoneum. Semi-quantitative in vivo animal experiments report uniform drug distribution in the abdominal cavity since the aerosol droplets are assumed to behave “gas-like” [4]. Furthermore, the increased intra-abdominal pressure in the shape of a capnoperitoneum of 12 mmHg C0_2_ counteracts the increased intratumoral pressure which amplifies the influx of drugs into the tumor tissue. Penetration depth into tumoral tissue is reported to be as deep as 500 to 600 μm with high tissue concentrations observed up to 1.70 μmol/g [5]—which is assumed to be higher than observed for liquid IPC. Yet, so far, PIPAC has never been compared to any liquid IPC treatment in a preclinical or clinical setting.

Recently published data report that the spatial drug distribution pattern of PIPAC is non-homogeneous. Furthermore, the aerosol delivered by the PIPAC micropump has a mean droplet size which is too large to distribute homogeneously. About 97.5 vol% of the delivered aerosol droplets are not submicronic and have not the physical properties to distribute gas-like [6]. Tissue areas located outside the aerosol spray jet of the PIPAC micropump only show a minimal drug exposure to the submicronic PIPAC aerosol [7].

In the clinical setting, a recently published phase II trial with doxorubicin and cisplatin in women suffering from recurrent platinum-resistant ovarian cancer delivered into the abdominal cavity as a pressurized aerosol endorsed the preclinical findings with a reported clinical benefit rate of 68% and a low incidence of severe side effects [8]. Similar findings are reported in patients suffering from platinum-resistant peritoneal carcinomatosis from colorectal cancer (pcCRC) treated with PIPAC and oxaliplatin in a compassionate use program for patients who did not qualify for cytoreductive surgery (CRS) and heated intraperitoneal chemotherapy (HIPEC) [9].

While first clinical results are encouraging, there remain a number of patients who show no response to PIPAC therapy or suffer an early disease relapse following an initial tumor regression after PIPAC therapy. In a clinical setting, it would crucial if one could increase the cytotoxic effect of PIPAC therapy by modulating different treatment parameters. However, the treatment parameters currently in use, i.e., intra-abdominal pressure, exposure time of the peritoneum to the pressurized aerosol, and temperature and dosage/concentration of the drugs delivered, have never been explored in detail and are solely based on assumptions and data derived from liquid IPC. Based on the above background data, we aimed to establish an in vitro PIPAC model to investigate any cytotoxic effect of the submicronic PIPAC aerosol and to explore a possible effect of different treatment parameters to modulate cytotoxicity.

Due to its technical design, the currently used PIPAC technology cannot be utilized in a small animal model of peritoneal carcinomatosis while a large animal model with a body size equivalent to the human body has never been established. Therefore, based on a previously established in vitro PIPAC model, colonic cancer cells were directly exposed to the PIPAC test atmosphere, a common model to assess cytotoxicity in aerosol science.

## Methods

### Cell cultures

The human colorectal cancer cell lines HCT8 were obtained from the Cell & Tumor Bank of the University Duisburg-Essen, Medical School. The HCT8 cell line is well characterized and frequently used in preclinical investigations since its resistant entity reveals highly metastatic rates in animal models, which mimic very well the clinical situation seen in peritoneal carcinomatosis [10–12]. Previous studies conducted in 3D cultures revealed no differences respecting 2D cultures. Chemoresistance was induced with etoposide which is very particular since it induces a cross-resistance behavior against several cytostatic drugs, including cisplatin and its derivatives (see Additional file 1). Both wild type (wt) and chemotherapy resistant type (rt) were maintained in Dulbecco’s modified Eagle’s medium (DMEM; PAN-BIOTech., No. P04-04510, PAN Biotech GmbH, Aidenbach, Germany), supplemented with 10% heat-inactivated fetal bovine serum, also purchased from PAN Biotech GmbH, 0.8 μl/ml of doxycycline, and incubated at 37 °C in a humidified 5% CO_2_/air atmosphere. Cells were seeded in 24-well plates at a density of 20,000 cells of HCT8 and 40,000 cells of HCT8^RETO^ (colon adenocarcinoma cell line resistant to etoposide) per well and incubated at 37 °C with 5% CO_2_. During the PIPAC experiments, the cell cultures medium was aspired, so that the cells were directly exposed to the test atmosphere. After the exposure period, the cell cultures were immediately covered with medium and the culture was continued for 48 hrs. At the end of the treatment 200 μl of MTT solution (5 mg/ml in PBS) were added into each well, followed by incubation at 37 °C for 3 h. The culture medium containing MTT was aspirated, and the formazan crystals formed were then solubilized with 200 μl DMSO for 30 min. Absorbance was measured at wave length 570 nm in a microplate reader (Tecan, Basel, Switzerland), and the percentage of proliferation was determined for all groups.

### Ex vivo PIPAC model and PIPAC procedures

The ex vivo PIPAC model has been previously described in detail [7]. In brief, the PIPAC model was placed in a water bath and kept at a constant temperature of 37 °C during the entire procedure. For each experiment, two 24-well plates (TPP techno Plastic Products AG, Trasadingen, Switzerland) were then placed at the bottom of the plastic box, laterally of the aerosol jet spray, and covered with a bilaterally open plastic tunnel to avoid direct exposure of the tumor cells to the aerosol jet of the MIP® (MIP®, Reger Medizintechnik, Rottweil, Germany). The plastic box was then tightly sealed, and the CO_2_ capnoperitoneum (Olympus UHI-3; Olympus, medical life science and industrial divisions, Olympus Australia, Notting Hill, Australia) was established and continued for the entire PIPAC procedure. Oxaliplatin (Teva GmbH, Radebeul, Germany) was first aerosolized (aerosol phase) and then applied onto the exposed tumor cells (exposure phase).

The clinical standard of PIPAC therapy for pcCRC is as follows: oxaliplatin (92 mg/m^2^ body surface at 23 °C) diluted in 150 ml glucose 5% is aerosolized in a capnoperitoneum of 12 mmHg at 36 °C [9]. The drug solution is delivered with a flow of 30 ml/min (Injektron 82 M, MedTron, Saarbrücken, Germany) to a patented micropump which delivers a polydisperse aerosol. After termination of the injection of the chemotherapy solution (aerosol phase), the peritoneal cavity is exposed for another 30 min (exposure phase) to the oxaliplatin aerosol. To investigate the role of the different treatment parameters on tumor cell cytotoxicity, the experiments were performed as follows.

### Cell culture exposure to test aerosol atmosphere

To test any cytotoxic effect of the PIPAC test atmosphere on medium free cell cultures, the cell cultures were exposed to an aerosol phase of 5 min with 150 ml glucose 5%, capnoperitoneum of 12 mmHg, 36 °C, and variable exposure phase of 0, 15, 30, and 45 min. Control group (CG): 150 ml glucose 5% but cell cultures exposed to PIPAC aerosol in medium.

#### Oxaliplatin dose

Aerosol phase of 5 min, exposure time of 30 min, and a constant capnoperitoneum of 12 mmHg at 36 °C. Oxaliplatin doses of 92, 138, and 184 mg diluted in 150 ml glucose 5% at 23 °C. CG: 150 ml glucose 5%.

#### Pressure

Aerosol phase 5 and 30-min exposure time with oxaliplatin (92 mg in 150 ml glucose 5% at 23 °C). Variable pressure of the capnoperitoneum: 5, 10, 15, and 20 mmHg at 36 °C. CG: PIPAC at atmospheric pressure.

#### Exposure time

Oxaliplatin (92 mg in 150 ml glucose 5%) at 23 °C and aerosol phase of 5 min in a constant capnoperitoneum of 12 mmHg at 36 °C. Variable exposure time: 15, 30, and 45 min, respectively. CG: no aerosol exposure time.

#### Temperature of the oxaliplatin solution

Oxaliplatin (92 mg in 150 ml glucose 5%) and aerosol phase and exposure time of 5 and 30 min with a 12 mmHg capnoperitoneum at 36 °C. Temperature of the oxaliplatin solution aerosolized: 27, 36, and 43 °C. CG: oxaliplatin delivered at 23 °C.

### Statistical analysis

All experiments were performed at least in triplicate. Each well was considered as a single value, corresponding to the subgroups. The Kruskal-Wallis one-way analysis of variance on ranks was used to compare independent groups. Probability (*p*) values were considered as follows: *p < 0.05; **p < 0.01; ***p < 0.001; ^#^ *p* > 0.05.

## Results

### Cell culture exposure to test atmosphere

The cell viability in the cell cultures directly exposed to the test PIPAC atmosphere (no medium) was similar to that observed for those covered with medium (submerged exposure). During the aerosol and exposure phase, the humidity in the PIPAC model remained at 100% with a constant temperature between 36 and 37 °C (data not shown).

### Oxaliplatin dose escalation

Compared to the CG, significantly higher cell toxicity was observed in all treatment groups (rt and wt cells; *p* < 0.001). In wt cells, any increase of oxaliplatin dosage progressively leads to significantly higher cell cytotoxic (*p* < 0.05).

**Fig. 1:**
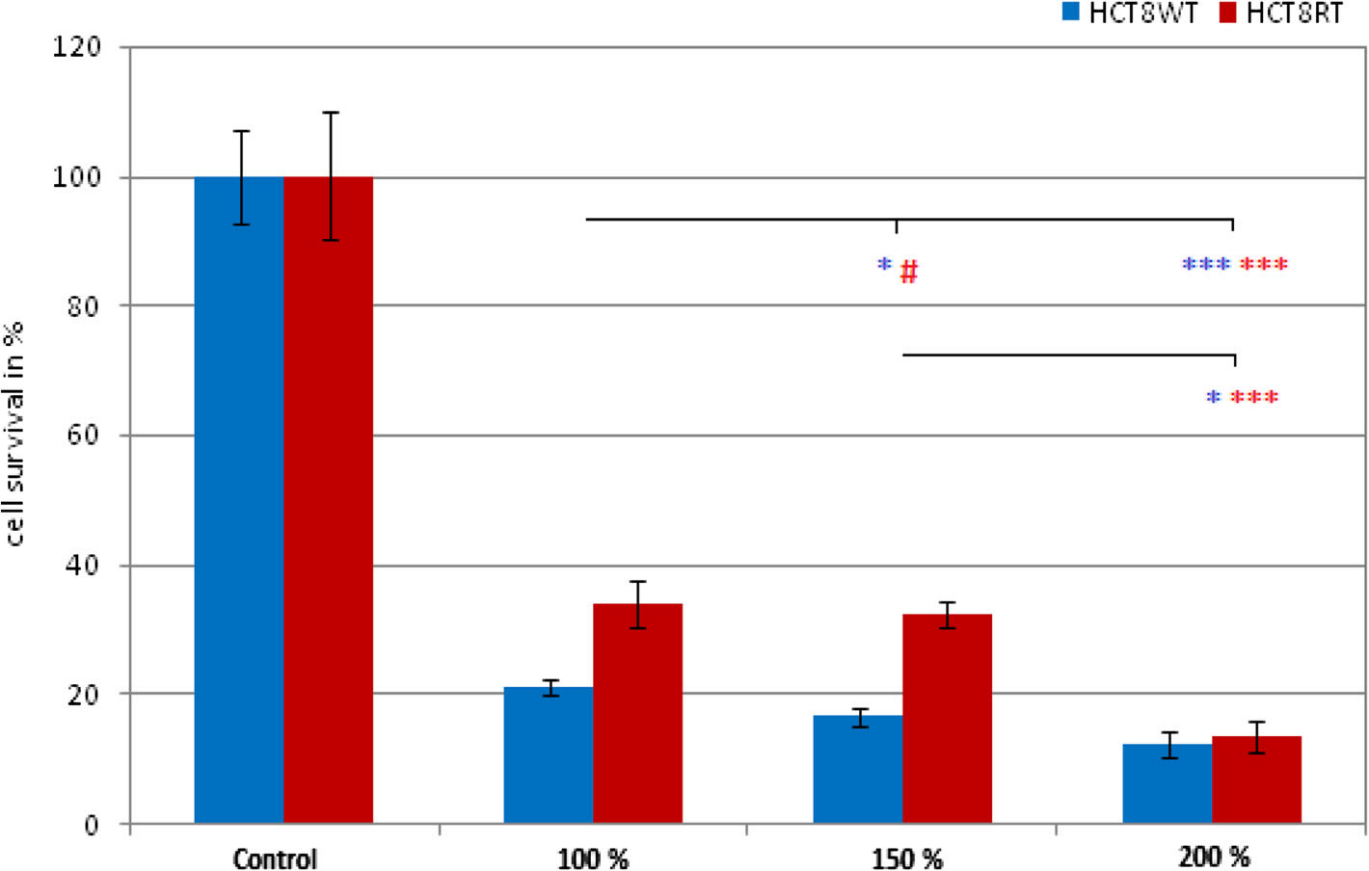
Effect of oxaliplatin dose escalation on cell toxicity. *Control* control group; 100% = 92 mg; 150% = 138 mg; 200% = 184 mg oxaliplatin. *p < 0.05; **p < 0.01; ***p < 0.001; ^#^ *p* > 0.05

However, this effect was not observed in rt cells comparing 92 vs. 138 mg (*p* > 0.05). Nevertheless, an increase from 138 to 184 mg resulted in higher tumor cell death (138 vs. 184 mg; *p* < 0.001). The results of dose escalation are summarized in Fig. 1.

### Effect of pressure

**Fig. 2:**
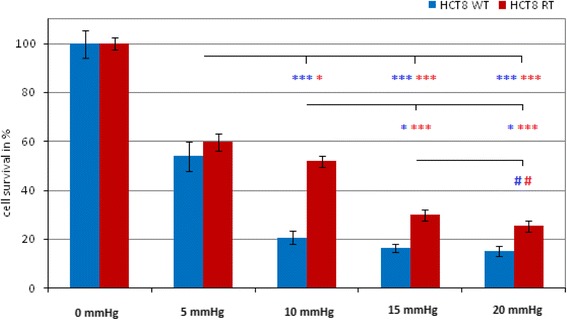
Effect of pressure on tumor cell cytotoxicity: 0 mmHg = control group; *p < 0.05; **p < 0.01; ***p < 0.001; ^#^ *p* > 0.05

Increase of pressure significantly affects cytotoxicity. A maximum was observed at a pressure of 15 mmHg since a further increase of pressure to 20 mmHg did not result in significantly higher cell toxicity (15 vs. 20 mmHg; *p* > 0.05). The effect of pressure is summarized in Fig. 2.

### Exposure time and temperature of the oxaliplatin solution

Standard PIPAC without additional exposure time served as CG. Additional exposure time of 15, 30, and 45 min did not affect the cytotoxic effect. The same observation was made with an increase of temperature of the oxaliplatin solution (27, 36, and 43 °C; data not shown).

## Discussion

One of the key elements of PIPAC therapy is the fact that the intra-abdominal drug distribution pattern is homogeneous since the aerosol would behave gas-like. However, this is in strong contrast to recent findings which report that the aerosol fraction which can distribute gas-like (submicron aerosol) is less than 3 vol% of the total amount of the chemotherapy delivered during PIPAC therapy [6]. This observation is in strong contrast to first clinical data about PIPAC therapy where up to 70% of patients suffering from chemoresistant end-stage peritoneal carcinomatosis show objective regression of PC. In our current in vitro PIPAC model, the submicron (gas-like) test aerosol showed cell cytotoxicity. This fact is even more important, since PIPAC therapy for colorectal peritoneal carcinomatosis uses only 20% of liquid IPC. Taken together, 3 vol% of submicron PIPAC aerosol out of only 20% of a liquid IPC dose induces cytotoxicity in multi-chemoresistant colonic cancer cells in our in vitro model. These findings have important implementations. First, our data are an additional argument to support the PIPAC concept. Second, if technical innovations can furthermore increase the fraction of submicron aerosol delivered during PIPAC therapy, the efficiency of this approach could probably be further increased.

In contrast to lung medicine and inhalation toxicology, there are so far no standardized models which would allow easy and reliable testing of different chemotherapy aerosol to treat peritoneal carcinomatosis. Our presented PIPAC model is a simple and easy testing tool which is based on the principle of air-liquid interface testing systems, where the cells are grown on a permeable membrane in contact with an underlying medium and the cells are directly exposed to a constant test atmosphere. However, the constant flow of the test atmosphere induces dry out effects of the cells and changes in the environmental cell milieu. In contrast to this, the PIPAC aerosol is delivered in a constant capnoperitoneum with no flow effects and a constant temperature and humidity of 100%. With that background, we exposed the cell cultures without any medium and observed that the cell viability after an exposure time of up to 50 min to a test atmosphere of glucose 5% remained intact. Therefore, we believe that our in vitro PIPAC aerosol exposure model is a first step to assess the toxic effect of different aerosols applied for possible PIPAC therapy without the disadvantages for cell cultures under submerged conditions.

Since the first clinical use of PIPAC, the possibility to further enhance the antitumor effect by an additional increase of the intra-abdominal pressure (IAP) >12 mmHg has never been investigated. Our current study shows that a progressive increase of the IAP results in significantly higher rates of tumor cell death in both wild and chemoresistant tumor cells. A further increase of IAP to 20 mmHg showed an additional yet not significant cytotoxic effect compared to 15 mmHg. However, we are aware of the fact that our in vitro cell experiments have some limitations and must be interpreted with caution with respect to in vivo pharmacokinetics. Nevertheless, our findings are in line with previous results obtained in animal models. Jacquet at al. have proposed to increase the IAP to counteract the elevated intra-tumoral pressure for improving the tissue penetration depth and tissue concentrations of doxorubicin for IPC. A significantly increased tissue concentration of doxorubicin at an IAP of 20 to 30 mmHg could be observed in a rat model [13]. Esquis and co-workers demonstrated in a rodent peritoneal carcinomatosis model that increased IAP of 22 mmHg for 1 h resulted in a significantly higher cisplatin penetration in tumoral tissue and improved survival of the animals [14] and even a moderate increase of IAP (18 mmHg) in healthy pigs showed significantly increased tissue concentrations for cisplatin [15, 16]. However, the cutoff range of increased IAP where pharmacological characteristics of IPC are optimized but with an acceptable additional risk of local and systemic complications due to the increased IAP is not known. Nevertheless, in clinical use, safe laparoscopic HIPEC procedures with an IAP up to 15 mmHg for palliating malignant ascites have been reported [17], and a capnoperitoneum of 20 mmHg can be safely performed in most cases [18]. Therefore, an increase of the IAP >15 mmHg may be a valuable option in patients who show no response to repetitive standard PIPAC therapy or suffer from a disease relapse shortly after successful PIPAC therapy. However, the established occupational health safety concept of PIPAC therapy has only been explored for an IAP of 12 mmHg [19].

In most centers, CRS and HIPEC for pcCRC are currently performed with oxaliplatin. Oxaliplatin dosage for PIPAC therapy in patients who do not qualify for CRS and HIPEC is currently 92 mg/m^2^ body surface diluted in 150 ml of glucose 5% [16]. Until today, a higher drug dosage for PIPAC therapy has not been investigated. Our first in vitro data on dose escalation found a significantly higher cytotoxic effect of PIPAC therapy in wild-type colonic cancer cells for 138 and 184 mg of delivered oxaliplatin. Increased drug dosage is assumed to amplify in tissue depth penetration and in tissue drug concentrations. Yet, a significantly increased cell death rate in chemotherapy-resistant tumor cells was only attained when the dose was doubled from 92 to 184 mg. With regard to our data, we view a dose escalation as a valuable tool in patients who show no response to standard PIPAC therapy regiments. Currently, a phase I dose escalation study for PIPAC with cisplatin and doxorubicin is running at our institution [20]. However, for oxaliplatin, dose finding studies are urgently needed.

To date, a wide variety in the duration (30 to 120 min) of intraperitoneal chemotherapy protocols (HIPEC) has been reported. In PIPAC therapy, the chemotherapy drug is aerosolized during 5 min with an additional exposure time of 30 min. In our current in vitro PIPAC model, additional exposure time showed no increase in the cytotoxic effect. Similar findings were reported by Jacquet et al. in a rat model. IPC with an increased IAP showed significantly higher tissue concentrations with its maximum already after 10 min of exposure time [13]. Studies with growth inhibition assays on human lung cancer cells show that dose escalation with increased concentrations of gemcitabine delivered as an aerosol enhanced the cytotoxic effect while prolonged exposure time did not [21]. Since aerosol has an optimum of surface area at the interface of the tumor, the influx of antineoplastic drugs into peritoneal and tumoral tissue seems to achieve high tissue drug concentrations already after a short exposure time. Thus, in a clinical setting, exposure time may be shortened resulting in reduced intervention time as well as minimized operative trauma to the patient while saving health care costs.

As the final parameter, a rise in drug temperature was analyzed with regard to a possible enhanced cytotoxic effect. Hyperthermia itself is known to induce cytotoxicity of malignant cells, augment the cytotoxic effect of different chemotherapeutic drugs such as doxorubicin and platin compounds, and increase the penetration depth of the chemotherapeutic drugs (reviewed in [22]). During PIPAC therapy, the intra-abdominal temperature cannot be increased since the abdominal cavity is a closed system. Theoretically, a local hyperthermia could be achieved by insufflation of heated C0_2_ (41–43 °C) prior to delivering PIPAC. However, during the procedure, no heated C0_2_ can be delivered anymore, and the temperature would rapidly decrease to levels of body temperature. Since data of aerosol therapy of the lung reported that an increase of temperature of the aerosolized liquid drug decreases the viscosity and surface tension leading to a reduced aerosol droplet size and a higher saturated vapor pressure [23]—thermodiffusion could additionally improve the aerosol deposition on the tumoral tissue and enhance drug influx. Based on these data, we explored whether an increase of the temperature of the aerosolized drug would be a possibly easy and cheap way to enhance the effectiveness of PIPAC therapy. However, our theoretical assumptions were not confirmed in our in vitro PIPAC model. Even at a temperature of 43 °C of the aerosolized chemotherapeutic liquids, no additional cytotoxic effect was explored. However, a recently published research article reported about the safety and feasibility of hyperthermic PIPAC (H-PAC) in an experimental setting in a swine model [24].

The theoretical assumptions on a possible benefit of hyperthermia were not confirmed in our in vitro PIPAC model. Even at a temperature of 43 °C of the aerosolized chemotherapeutic liquids showed no additional cytotoxic effect.

## Conclusions

Aware of the limitations of our in vitro experiments, we aimed to give an insight on the cytotoxic effect of PIPAC as well as have an outlook on how clinical parameters may be modified in the near future. Increased IAP and drug dose escalation amplify the cytotoxic effect, while prolonged exposure time to the aerosol shows no amplification. Our data are in line with previous reports from in vivo animal and in vitro cell experiments. However, further basic research is required to increase the evidence of our findings.

When PIPAC therapy was first applied to patients, the treatment parameters were mostly based on assumptions, limited experimental data, and personal expertise. However, we believe that in patients who show no response to standard PIPAC therapy or who show tumor regression after successful PIPAC therapy, an increase of IAP to 15 to 20 mmHg as well as dose escalation might be a valuable clinical option.

## Additional file

Additional file 1: Details about the induction of chemoresistance and characteristaion of the cells. (DOC 6237 kb)

## Abbreviations

CG: Control group
CO_2_: Carbon dioxide
CRS: Cytoreductive surgery
DMSO: Dimethyl sulfoxide
HIPEC: Heated intraperitoneal chemotherapy
IAP: Intra-abdominal pressure
IPC: Intraperitoneal chemotherapy
MTT: MTT 3-(4,5-Dimethylthiazol-2-yl)-2,5-Diphenyltetrazolium Bromide
PBS: Phosphate-buffered saline
PC: Peritoneal carcinomatosis
pcCRC: Peritoneal carcinomatosis from colorectal cancer
PIPAC: Pressurized intraperitoneal aerosol chemotherapy
rt: Chemo-resistant type human colonic cancer cell line
RT: Room temperature (23 °C)
wt: Wild-type human colonic cancer cell line
