# Erratum to: Cytotoxic effect of different treatment parameters in pressurized intraperitoneal aerosol chemotherapy (PIPAC) on the in vitro proliferation of human colonic cancer cells

**DOI:** 10.1186/s12957-017-1155-y

**Published:** 2017-05-02

**Authors:** Veria Khosrawipour, David Diaz-Carballo, Ali-Haydar Acikelli, Tanja Khosrawipour, Thomas Albert Falkenstein, Dan Wu, Jürgen Zieren, Urs Giger-Pabst

**Affiliations:** 1 Department of General Surgery and Therapy Center for Peritonealcarcinomatosis, St. Mary’s Hospital Herne, Ruhr University of Bochum, Herne, Germany; 2 Basic Research Laboratory Department of Surgery, St. Mary’s Hospital Herne, Ruhr University of Bochum, Herne, Germany; 3 Department of Hematology and Medical Oncology, St. Mary’s Hospital Herne, Ruhr University Bochum, Herne, Germany

## Erratum

Upon publication of the original article [1], it was noticed that the author’s name “Ali-Haydar Acikelli” was incorrectly given as “Acikelli Ali-Haydar”. This error was due to an error in the XML tagging of the author’s Family and Given names. This has now been acknowledged and corrected in this erratum.
